# ASO Author Reflections: The Magic of Clinical Research—The Student-Led PATRONUS Study Unveils Two Patient-Reported Outcome Measures for Use in Surgical Oncology

**DOI:** 10.1245/s10434-021-09648-x

**Published:** 2021-02-22

**Authors:** The CHIR-Net SIGMA Study Group, André L. Mihaljevic

**Affiliations:** Department of Surgery, University Hospital Heidelberg, Heidelberg, Germany

## Past

Patient-reported outcome (PRO) measures are important in clinical trials, routine care, and quality improvement programs to capture the “personal assessment of the burden and impact of a malignant disease and its treatment”.^1^ Recently, two new PRO measures have been described to capture cancer- and treatment-related symptoms and health-related quality of life: (1) the PRO version of the Common Terminology Criteria for Adverse Events (PRO-CTCAE^TM^) to measure symptoms;^2^ and (2) the computed adaptive testing version of the European Organisation for Research and Treatment of Cancer’s (EORTC’s) quality-of-life questionnaire (CAT EORTC QLQ-C30).^3^ These two PRO measures have the potential to be tailored to individual patients, cancer types, or treatment groups, but their implementation in patients undergoing cancer surgery has not yet been evaluated.

## Present

Overall, 303 patients undergoing major abdominal cancer surgery were enrolled at 15 sites in a prospective cohort study by a student-led clinical research network (CHIR-*Net* SIGMA).^4^ More than 100 medical students cooperatively conducted, analyzed, and reported this study under the supervision of academic surgeons. Patients reported PRO data up to 6 months postoperatively using electronic versions of the CAT EORTC QLQ-C30 and the PRO-CTCAE^TM^. Twelve core cancer symptoms were assessed via the PRO-CTCAE^TM^.^5^ Postoperative morbidity was recorded according to the Dindo–Clavien classification. PRO measurements were accepted by patients undergoing major abdominal surgery, as data completeness was > 80% in the immediate postoperative period. At 3–6 months postoperatively, no PRO-CTCAE symptoms differed significantly to baseline, except diarrhea. Patients reported higher ‘social functioning’ (*p* = 0.021) and overall quality-of-life scores (*p* < 0.05) 6 months after cancer surgery compared with the baseline level. There was a lack of correlation between postoperative complications or death and any of the PRO items evaluated.

## Future

Our study showed the feasibility of using the CAT EORTC QLQ-C30 and the PRO-CTCAE^TM^ in surgical oncology. These two new PRO tools are attractive for wider application in surgical oncology as they can be tailored to specific needs and situations, and can therefore allow a more personalized PRO assessment. Furthermore, both tools have already been employed in medical oncology and palliative care^3^ and can therefore be used to assess total cancer and treatment burden from the patients’ perspective along an entire healthcare pathway.^6^ Future studies in more specific subgroups using disease-specific PRO items are needed to elucidate the prognostic value of the two PRO measures regarding postoperative morbidity and mortality. PATRONUS demonstrated the feasibility of student-led clinical research and uncovered important aspects for improvement in future research-based learning projects in medicine.
